# Efficient Structure from Motion for Large-Size Videos from an Open Outdoor UAV Dataset

**DOI:** 10.3390/s24103039

**Published:** 2024-05-10

**Authors:** Ruilin Xiang, Jiagang Chen, Shunping Ji

**Affiliations:** School of Remote Sensing and Information Engineering, Wuhan University, Wuhan 430079, China; xiang_ruilin@whu.edu.cn (R.X.); chenjiagang2015@whu.edu.cn (J.C.)

**Keywords:** structure from motion, SLAM, unmanned aerial vehicle, large-size videos, keypoint adjustment

## Abstract

Modern UAVs (unmanned aerial vehicles) equipped with video cameras can provide large-scale high-resolution video data. This poses significant challenges for structure from motion (SfM) and simultaneous localization and mapping (SLAM) algorithms, as most of them are developed for relatively small-scale and low-resolution scenes. In this paper, we present a video-based SfM method specifically designed for high-resolution large-size UAV videos. Despite the wide range of applications for SfM, performing mainstream SfM methods on such videos poses challenges due to their high computational cost. Our method consists of three main steps. Firstly, we employ a visual SLAM (VSLAM) system to efficiently extract keyframes, keypoints, initial camera poses, and sparse structures from downsampled videos. Next, we propose a novel two-step keypoint adjustment method. Instead of matching new points in the original videos, our method effectively and efficiently adjusts the existing keypoints at the original scale. Finally, we refine the poses and structures using a rotation-averaging constrained global bundle adjustment (BA) technique, incorporating the adjusted keypoints. To enrich the resources available for SLAM or SfM studies, we provide a large-size (3840 × 2160) outdoor video dataset with millimeter-level-accuracy ground control points, which supplements the current relatively low-resolution video datasets. Experiments demonstrate that, compared with other SLAM or SfM methods, our method achieves an average efficiency improvement of 100% on our collected dataset and 45% on the EuRoc dataset. Our method also demonstrates superior localization accuracy when compared with state-of-the-art SLAM or SfM methods.

## 1. Introduction

Modern unmanned aerial vehicles (UAVs) equipped with cameras have become crucial in several fields, such as surveying and mapping, geographic information systems (GIS), and digital city modeling. To achieve accurate localization and create 3D representations of real-world scenes, techniques like image or video-based structure from motion (SfM) and visual simultaneous localization and mapping (VSLAM) are utilized [1,2,3,4,5,6,7,8,9,10]. However, it is important to note that there is a relatively limited amount of research on large-size video-based SfM specifically designed for outdoor UAVs. On the one hand, a mainstream UAV camera has already reached a resolution up to 20 megapixels, thus providing more detailed information for all kinds of applications. However, the widely-used video datasets [11,12,13,14,15] provide a resolution below 1 megapixel. On the other hand, there is limited research on how to combine SfM and VSLAM for large-size video-based localization. For large-size videos, current video-based SfM methods extract keyframes from videos usually based on simple empirical rules, for example, Kurniawan et al. [16] performed SfM on the keyframes extracted from videos simply according to the overlap rate of images, instead of a more sophisticated VSLAM method, to achieve 3D terrain reconstruction. In fact, VSLAM designed for continuous image processing inherently suits video data better. To process large-size videos in real-time, SLAM systems estimate camera poses and build maps on downsampled images, which is more efficient but results in lower localization accuracy than SfM methods. Some researchers [17,18] have attempted to utilize VSLAM to assist the SfM method for reconstruction. However, these methods only utilize estimated camera poses from a SLAM system. In fact, reusing feature extraction, matching, and keyframe covisibility graph results from SLAM can significantly reduce the computational cost for large-size video processing.

In this paper, we propose an efficient SfM pipeline designed to process high-resolution aerial videos. Additionally, we introduce a new outdoor UAV video dataset comprising images with a resolution of 3840 × 2160 pixels. Our approach maximizes the usefulness of initial outcomes provided by a speedy VSLAM system and incorporates a constrained bundle adjustment (BA) as a singular backend refinement step. The pipeline unfolds in the following steps. Initially, we subject the downsampled video data to a VSLAM system, which serves multiple purposes, including selecting keyframes and keypoints, as well as establishing preliminary camera poses and 3D scene structures. Secondly, to optimize the efficiency of the pipeline, we employ a coarse-to-fine two-step keypoint adjustment (TS-KA) method with rotation invariants, which adjusts the positions of matched keypoints projected onto the original high-resolution images instead of re-matching new feature points. This adjustment process begins by roughly aligning keypoint positions using normalized cross-correlation (NCC) [19]. Following the rough alignment, we apply direct image alignment [20] within a learned dense feature space to further refine matched points up to sub-pixel accuracy. Finally, the global bundle adjustment takes the initial camera poses from the VSLAM system as inputs and integrates a rotation averaging strategy [21]. Optionally, ground control point (GCP) constraints can be included to retain high-accuracy poses and 3D scene points at a centimeter-level precision.

The contributions of this paper are summarized as follows. (1) Efficient SfM pipeline. We propose an efficient pipeline specifically designed to process large-size aerial videos. By leveraging the strengths of a rapid VSLAM system and incorporating refined adjustment steps, our pipeline achieves impressive accuracy and efficiency in pose localization of video sequences. (2) Two-step keypoint adjustment (TS-KA) strategy. The novel strategy refines the positions of keypoints matched in downsampled images up to sub-pixel accuracy on the original high-resolution images. (3) High-resolution UAV video dataset. We provide a high-resolution UAV video dataset and supply high-accuracy GCPs to facilitate evaluation. This dataset fills a gap in the current availability of outdoor high-resolution video datasets for SLAM or SfM research.

## 2. Related Work

### 2.1. Unstructured, Sparsely Sampled Collection

Early works laid the foundation for internet photo collections [22]. Inspired by these works, reconstruction systems for increasingly high-resolution photo collections have been developed [15,23]. These methods can be classified into incremental SfM, global SfM, and hybrid SfM, based on the manner in which camera poses are estimated. Currently available open-source incremental SfM algorithms, such as Bundler [1], VisualSfM [2], and COLMAP [3,24,25], provide a solid foundation for SfM research. Mainstream global SfM methods [4,5,26,27] estimate all camera poses and perform a global BA to refine the camera poses and reconstruction scene, resulting in better scalability and efficiency. Rotation averaging [28,29,30,31] estimates all camera rotations from pairwise relative rotations, while translation averaging [32,33,34] calculates the translation of each camera pose. However, the latter may fail to estimate correct camera centers when the camera moves collinearly [4,5].

Indeed, these methods focusing on unordered, sparsely sampled images face challenges when dealing with coherent, densely sampled data. This difficulty arises from frame-wise matching and triangulation with very short parallax, which can result in high computation loads and unreliable geometric structures.

### 2.2. Coherent, Densely Sampled Collection

This type of study addresses continuous feature tracking and mapping on coherent, densely sampled image sequences. Specifically, VSLAM methods have been developed to estimate camera trajectories and reconstruct scene structures from video streams in real time [7,8,9,10,35]. However, these methods often prioritize speed and, as a result, face limitations when processing large-size high-resolution images. This restriction hampers their ability to produce fine-grained high-quality reconstructions.

Over the years, SfM methods have also been developed specifically for densely sampled image sequences or videos. For example, Shum et al. [36] introduced the concept of “virtual keyframes” in a hierarchical SfM approach to enhance efficiency. Resch et al. [37] proposed multiple SfM techniques based on the KLT tracker and linear camera pose estimation [38] for large-scale videos. Leotta et al. [39] accelerated feature tracking for aerial videos by exploiting temporal continuity and planarity of the ground. More recently, a deep learning-based approach [40] was proposed to select appropriate keyframes for videos. To resolve ambiguity arising from repetitive structures, Wang et al. [41] proposed a track-community structure to segment the scene. Gong et al. [42] proposed to disambiguate scenes in SfM by prioritizing pose consistency over feature consistency. However, it should be noted that these methods may rely on fixed camera calibration and could encounter significant drift issues in scenes without a loop. Different from these methods, our work proposes a hybrid SfM solution that combines the advantages of global SfM and feature-based VSLAM methods.

### 2.3. Keypoint Adjustment

Recently, there has been an increased focus on developing local search-based methods to enhance the efficiency and accuracy of keypoint matching. These methods employ both handcrafted [43,44] and learned features [45,46,47] to establish more accurate correspondences between keypoints. For example, Taira et al. [48] presented a method that achieves dense correspondence through a coarse-to-fine matching process using VGG-16 [49]. Li et al. [50] employed a dual-resolution approach to achieve reliable and accurate correspondences. Zhou et al. [51] proposed a detect-to-refine method, where initial matches are refined by regressing pixel-level matches in local regions. However, it should be noted that these methods [48,50,51] are primarily optimized for stereo pairs and may not be directly applied for multiple views.

In order to enhance the quality of multi-view keypoints for downstream tasks like SfM, Dusmanu et al. [52] incorporated a geometric cost with optical flow. However, this method has limitations in terms of accuracy and scalability for large scenes. Lindenberger et al. [20] addressed the alignment of keypoints by utilizing feature-metric representation to jointly adjust feature matches across thousands of images. However, this method suffers from a limited range of adjustment and may become less accurate when dealing with images exhibiting significant viewpoint changes. To address these challenges, we introduce an efficient two-step matching approach that takes into account errors in initial matching at a lower resolution and effectively handles large viewpoint changes.

## 3. Proposed Method

### 3.1. System Overview

**Figure 1:**
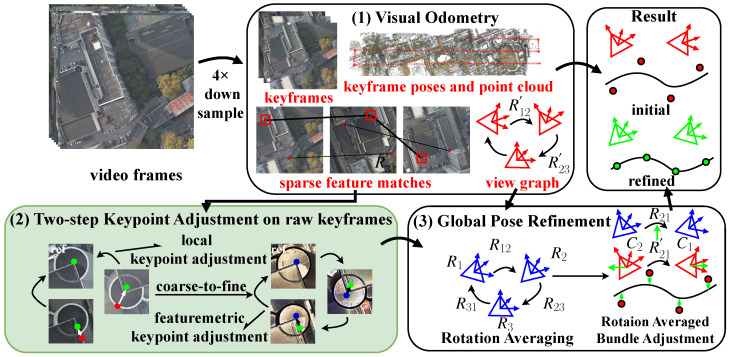
System overview. (1) The initial scene structure is obtained from 4× downsampled video using visual odometry. (2) Keypoints are refined on full-resolution keyframes by a two-step keypoint adjustment method. red: original matching points; green: matching points after coarse keypoint adjustment; blue: matching points after sub-pixel refinement. (3) Global rotation is obtained by rotation averaging, and scene structure is finally refined using rotation-averaged bundle adjustment.

We introduce a novel SfM pipeline that efficiently selects appropriate keyframes and calculates camera poses by utilizing rich information from high-resolution, high-frame-rate videos. As depicted in Figure 1, our proposed pipeline comprises three main steps.

In the first step, we begin by downsampling the original high-resolution video to improve efficiency. Then, we estimate the initial camera poses and select keyframes using visual odometry on the downsampled video. The output of this step includes three components: a set of *N* keyframes denoted as $I=\{I_{1},\ldots ,I_{N}\}$ along with their poses $T_{I_{i}}^{W}=\left\{\left(R_{I_{i}}^{W},t_{I_{i}}^{W}\right)\in \mathrm{SE}\left(3\right)\right\}$, M sparse world points $P=\{P_{l}^{W}\in R^{3}\}$ paired with their corresponding 2D keypoints $\left\{p_{u}\right\}$, and a view graph (VG) G={V,E} with absolute rotation ($R_{I_{i}}^{W}$) as vertices and relative rotation ($R_{I_{i}}^{I_{j}}$) as edges for image pairs ($I_{i},I_{j}$).

In the second step of the pipeline, we upsample the keypoints obtained from visual odometry to match the original resolution of the keyframes. To achieve sub-pixel accuracy, we employ a two-step keypoint adjustment method called TS-KA. TS-KA refines the position of the upsampled keypoints in a coarse-to-fine strategy. Initially, we utilize the NCC algorithm [19] to roughly adjust the keypoint positions considering view angle changes. Then, we introduce feature-metric optimization for further refinement.

Moving on to the third step, we perform rotation averaging on the VG obtained in the first step. This helps us estimate the global rotation of all keyframes. The obtained global rotation will be integrated into BA as a regularization measure, reducing cumulative errors. Then, we refine the camera intrinsic parameters, keyframe poses, and sparse point cloud coordinates through rotation-averaged BA. To handle outliers, we incorporate a reprojection error threshold to filter them out. Additionally, we enhance trajectory accuracy at the centimeter-level by including GCPs in the BA process.

### 3.2. Initial Pose Estimation

We utilize visual odometry for both keyframe selection and initial scene reconstruction. Given the high-resolution aerial video used in this study, we initially downsample the raw video by a factor of 4. This downsampling step ensures real-time initial camera trajectory estimation. Visual odometry involves the detection and tracking of distinctive features in consecutive camera frames. By matching these features, it estimates the camera’s relative motion and selects keyframes that represent significant viewpoints. In our proposed pipeline, we leverage the widely used OpenVSLAM for the initial camera pose estimation. OpenVSLAM includes three modules: tracking, local mapping and loop closing. The tracking module is primarily responsible for estimating the camera’s pose in real time. This module estimates the camera’s position and orientation by extracting and tracking feature points from consecutive video frames. It also determines whether to incorporate the current frame as a keyframe into the map based on specific rules. The local mapping module focuses on building and maintaining the map. It uses feature points from keyframes, creates new map points via triangulation, and performs local optimization of the map’s structure to enhance its accuracy. The loop closing module detects and handles loop closures. By recognizing revisited images and aligning them with previous map data, the module corrects cumulative navigational errors. More details can be found in [8].

### 3.3. Two-Step Keypoint Adjustment

As the matched points in VSLAM are obtained from 4× downsampled images with limited precision in point coordinates, it is necessary to adjust the keypoint coordinates to sub-pixel accuracy on the original resolution. Inspired by the work of the keypoint adjustment method in Pixel-SfM [20], we propose a simple yet powerful two-step keypoint adjustment approach TS-KA.

#### 3.3.1. Coarse Keypoint Adjustment

**Figure 2:**
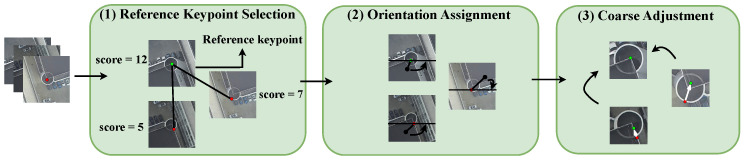
Coarse keypoint adjustment. (1) The reference keypoint is selected as the one having the highest score. (2) Each keypoint is assigned with a consistent orientation. (3) Best matching points (green ones) are obtained using NCC.

The coarse keypoint adjustment in Figure 2 aims to refine the keypoints within the given search area by utilizing a rotation-invariant similarity measure. This adjustment allows for accurate keypoint refinement over a large range. The first step involves determining the reference keypoint, $p_{r}$, within a track, $\left\{p_{i}\right\}$ (*i* = 1, …, *N*). A track refers to a collection of *N* keypoints corresponding to the same 3D world point. We calculate the accumulated matching scores for each keypoint in the track, and $p_{r}$ is selected as the reference keypoint with the highest accumulated matching score. The remaining points in the track are then adjusted and matched to $p_{r}$. Second, we assign a consistent orientation to each keypoint based on local image characteristics; the keypoint can be represented relative to this orientation, thus ensuring invariance to image rotation. For a pixel, p, within the search region of a keypoint, $p_{i}$, we compute the gray centroid, $p_{c}$, of its NCC window. The NCC window is a circular window with a radius of 15 pixels here. To achieve orientation invariance, we rotate the NCC window based on the angle between $pp_{c}$ and $p_{r}p_{rc}$, where $p_{rc}$ represents the gray centroid of $p_{r}$. Finally, we obtain the best matching points through NCC matching.

#### 3.3.2. Sub-Pixel Refinement

The coarse keypoint adjustment primarily achieves feature matching accuracy at the pixel level. To meet the accuracy requirements of various downstream tasks, it is often necessary to refine keypoints to sub-pixel accuracy. For this purpose, we introduce the feature keypoint adjustment (FKA) method [20]. We first extract a dense feature map of 16 × 16 patches centered on the keypoints by S2DNet [53], and then we treat the refinement of the keypoints, M(l), in a track belonging to the same landmark, *l*, as an energy minimization problem, as follows:

(1) $$E_{FKA_{l}}=\sum_{\left(a,b)\in M(l\right)}\omega_{ab}∥F_{i(a)}\left[p_{a}\right]-F_{j(b)}\left[p_{b}\right]{)∥}_{\gamma }$$

where $\omega_{ab}$ represents the confidence between matched points $p_{a}$ and $p_{b}$, according to the similarity of the local features. F[.] represents the feature map.

It should be noted that the original FKA [20] lacks a coarse adjustment step, which can result in numerous incorrect adjustments. To address this limitation, we have incorporated a coarse adjustment step in our approach. We also add a constraint $∥p_{best}^{cf}-p_{best}^{c}∥<K$, where $p_{best}^{cf}$ denotes the position of the keypoint after fine adjustment, and *K* is set to be lower than the radius, *r*.

### 3.4. Global Pose Refinement

The trajectory derived from visual odometry often suffers from the drift accumulation problem, leading to significant deviations from the true trajectory. Inspired by [21], in order to enhance the precision of camera pose estimation, we incorporate the global camera pose obtained through rotation averaging as a regularizer into the BA process. Furthermore, when available, we include the GCPs in the BA equations.

#### 3.4.1. Rotation Averaging

Rotation averaging (RA) is a method utilized for estimating global camera poses by simultaneously considering pairwise relative poses. The global rotation is computed by minimizing the cost function:

(2) $$\min_{R_{I_{1}}^{W},\cdots {,R}_{I_{N}}^{W}}\sum_{(I_{i},I_{j})\in E}d^{2}\left(R_{I_{i}}^{I_{j}},R_{I_{j}}^{W^{T}}R_{I_{i}}^{W}\right)$$

where $d^{2}$ represents the Euclidean norm. However, RA [21] is sensitive to outliers, which may result in inaccurate estimates.

Before performing RA, it is necessary to construct a view graph with edges being pairs of matched images. To avoid starting image matching from scratch, we leverage the co-visibility data derived from VO, as outlined in Section 3.2, transforming it into a view graph with candidate edges. Then, we assign higher weight values to edges with more visible points and a more uniform distribution of matches. Concurrently, we prune edges within a view graph under the following conditions: (1) the number of matches falls short of the predetermined threshold, $N_{m}$; and (2) the angular error for a given edge, denoted as $R_{I_{i}}^{I_{j}}$, is below a specified threshold, σ, as delineated by the following formula:

(3) $$d^{2}\left(R_{I_{j}}^{I_{i}}R_{I_{k}}^{I_{j}}R_{I_{i}}^{I_{k}},I_{3}\right)\leq \sigma$$

$I_{3}$ represents the 3 × 3 identity matrix. σ is set as 0.01.

#### 3.4.2. Rotation Averaged Bundle Adjustment

Rotation averaged BA is conducted to optimize camera poses, 3D points, and camera intrinsics. Since the observations are independent, the trajectory estimated by RA does not accumulate errors. Therefore, it can serve as a regularizer in BA to mitigate drift in the initial trajectory. The objective function for this optimization is as follows:

(4) $$\sum_{I_{i}\in I}\sum_{P_{l}^{W}\in P}\rho \left({∥r_{i,l}∥}^{2}\right)+\sum_{(I_{i},I_{j})\in E}\omega_{i,j}\left({∥{r^{\prime }}_{i,j}∥}^{2}\right)$$

where ρ(·) is the loss function, and, in this paper, the huber loss function $\rho \left(x\right)=\frac{x^{2}}{x^{2}+\delta^{2}}$ is used. $\omega_{i,j}$ represents the weight value for the known rotation term, ${r^{\prime }}_{i,j}$. The objective divides into two terms, explained as follows:

- Reprojection term: this term represents the reprojection error corresponding to all tie points in bundle adjustment, as follows:
  (5) $$r_{i,l}=\;\pi \left(R_{I_{i}}^{W}P_{l}^{W}+t_{I_{i}}^{W},\;C\right)-p_{i,l}$$
  where C represents the intrinsic matrix, and $P_{l}^{W}$ is the 3D coordinate of a world point. Additionally, GCPs can be included in the reprojection term, as follows:
  (6) $$P_{l}^{W}=s_{G}^{W}R_{G}^{W}P_{l}^{G}+t_{G}^{W}$$
  where $P_{l}^{G}$ denotes the position of GCP in the geodetic coordinate, and $R_{G}^{W}$, $t_{G}^{W}$, and $s_{G}^{W}$ represent the rotation matrix, the translation, and the scale between the world and geodetic coordinates. The centimeter-level accuracy trajectory can be obtained by introducing the GCP term into BA.
- Known rotation term: this term is used as a regularizer to reduce the accumulated error, which is given by:
  (7) $$r_{i,j}^{\prime }=\log\left(\hat{R_{I_{i}}^{I_{j}}}R_{I_{i}}^{W^{T}}R_{I_{j}}^{W}\right)$$
  where log is logarithm mapping from the special orthogonal group SO(3) to Lie algebra so(3). $\hat{R}$ and R denote estimated and global rotation, respectively.

## 4. Experiment

### 4.1. Dataset and Metrics

#### 4.1.1. Dataset

**Figure 3:**
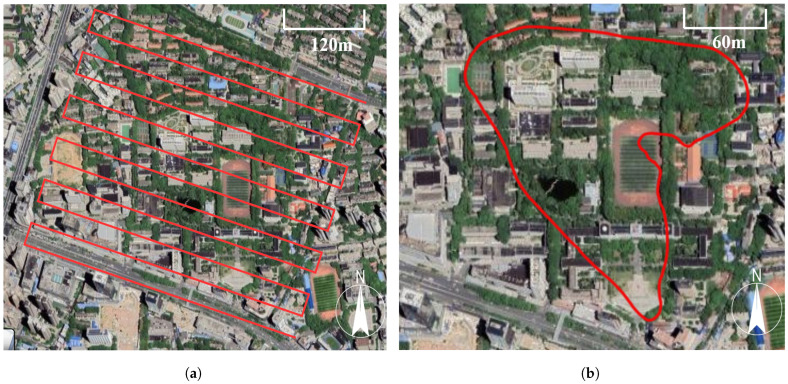
The visualization of dataset. (**a**) Regular scene. (**b**) Irregular scene. Red lines represent the trajectory of the drone.

The dataset consists of two aerial video sequences captured using the DJI M300 RTK drone with the DJI P1 camera, both manufactured by DJI in Shenzhen, China. Figure 3 illustrates the two sequences: one with a regular strip configuration and the other with an irregular configuration. These videos were recorded at the Informatics Department of Wuhan University at an altitude of 200 m. The recording frequency was set at 60 frames per second (fps), with a resolution of 3840 × 2160 pixels. The average ground resolution achieved was 0.03 m.

The regular sequence is a 1379-second video that contains evenly distributed air strips across the area. The between-strip overlapping is set at a degree of 40%. The coverage area of this sequence is 860 × 460 $m^{2}$ and consists of buildings, trees, and playgrounds. On the other hand, the irregular sequence is a 345-s video that follows a heart-shaped loop trajectory. This sequence includes wooded areas, buildings, and a lake, with numerous texture-repeated regions, making it more challenging compared with the regular sequence.

Additionally, we collected 16 GCPs that are evenly distributed throughout the dataset area. Some of these GCPs were utilized to compute a high-accuracy trajectory, while the remainder served as checkpoints to assess the accuracy of the trajectory. The GCPs were measured using a high-accuracy GPS receiver and processed to achieve a localization accuracy of 9.0 mm.

#### 4.1.2. Metrics

We use check points error (CPE) and absolute trajectory error (ATE) for evaluation.

- Check points error: the accuracy of triangulation is evaluated by utilizing surveyed points called check points (CPs) that were not used for georeferencing. Given a check point with coordinate ${\tilde{P}}_{l}=\left\{{\tilde{P}}_{l}\left(x\right),{\tilde{P}}_{l}\left(y\right),{\tilde{P}}_{l}\left(z\right)\right\}$, the root mean square errors (RMSEs) for plane ($\delta_{xy}$), elevation ($\delta_{z}$), and pixel ($\delta_{p}$) in terms of *m* CPs are evaluated as follows:
  (8) $$\begin{array}{c} \sigma_{xy}=\frac{1}{m}\sum_{l}^{m}\sqrt{{\left({\tilde{P}}_{l}\left(x\right)-P_{l}\left(x\right)\right)}^{2}+{\left({\tilde{P}}_{l}\left(y\right)-P_{l}\left(y\right)\right)}^{2}},\\ \sigma_{z}=\frac{1}{m}\sum_{l}^{m}\sqrt{{\left({\tilde{P}}_{l}\left(z\right)-P_{l}\left(z\right)\right)}^{2}},\;\sigma_{p}=\frac{1}{mN}\sum_{i}^{N}\sum_{l}^{m}∥r_{i,l}∥ \end{array}$$
- Absolute trajectory error: ATE is utilized to assess the drift in the position and rotation of the estimated trajectory. The estimated trajectory has been aligned with the ground truth trajectory using Umeyama’s method [54], resulting in aligned poses represented as $\left\{T_{I_{i}}^{W}\right\}=\left\{\left(R_{I_{i}}^{W},t_{I_{i}}^{W}\right)\right\}$. RMSEs for the position ($\delta_{pos}$) and rotation ($\delta_{rot}$) are evaluated as follows:
  (9) $$\begin{array}{c} \sigma_{\mathrm{pos}\;}=\frac{1}{N}∥t_{I_{i}}^{W}-\tilde{t_{I_{i}}^{W}}∥,\;\sigma_{\mathrm{rot}\;}=\frac{1}{N}\sum_{I_{i}\in I}d^{2}\left(R_{I_{i}}^{W},\tilde{R_{I_{i}}^{W}}\right) \end{array}$$

### 4.2. Results

In our method, the video input is set to 6 fps, and a downsampling rate of 4× is applied. The search radius for the two-step keypoint adjustment is set to 20 pixels. In contrast, the other methods utilize the original-scale videos as input. However, for methods like COLMAP and Theia that require temporally sampled keyframes, we employ two strategies. One strategy involves sampling the video images every one second. The other strategy involves using our method, as described in Section 3.2, which entails applying OpenVSLAM on the 4× downsampled videos to obtain keyframes.

We evaluate our method against the incremental SfM methods, namely COLMAP [3] and Theia [4], as well as the VSLAM method, OpenVSLAM [8], on the collected dataset, considering scenarios both with and without GCPs.

Performance on our collected dataset: As shown in Table 1, our pipeline has demonstrated significant improvements in accuracy when compared with COLMAP [3], Theia [4], and OpenVSLAM [8]. Specifically, in the regular sequence, our method outperforms the second-best method, OpenVSLAM, with improvements of 4.8 cm in $\delta_{xy}$ (a relative improvement of 137%), 1.7 cm in $\delta_{z}$ (a relative improvement of 40%), 0.12 pixels in $\delta_{p}$ (a relative improvement of 7%), as well as 0.7 m in $\delta_{pos}$ (a relative improvement of 175%), and 0.25° in $\delta_{rot}$ (a relative improvement of 178%).

Our method also exhibits significant improvements over other methods in all metrics for the irregular sequence. Compared with OpenVSLAM [8], the second-best performer, our method achieves improvements of 0.7 cm in $\delta_{xy}$ (a relative improvement of 14%), 6.2 cm in $\delta_{z}$ (a relative improvement of 293%), and 0.82 pixels in $\delta_{p}$ (a relative improvement of 103%). Additionally, our method demonstrates improvements of 0.24 m in $\delta_{pos}$ (a relative improvement of 48%) and 0.26° in $\delta_{rot}$ (a relative improvement of 96%). These results indicate that our pipeline effectively enhances reconstruction robustness and yields more accurate scene structure.

It is worth noting that when COLMAP [3] and Theia [4] are initialized with our selected keyframes, the accuracy improves across all metrics in both sequences against using per second sampling. This suggests that SLAM-based methods can effectively provide keyframes for subsequent SfM methods.

Table 1 presents a comparison of the efficiency of different methods. Our method requires the least amount of time compared with other methods, yielding a remarkable 200% enhancement over COLMAP [3], a 100% to 200% enhancement over OpenVSLAM [8], and a 50% to 100% improvement over Theia [4] for the regular sequence.

Performance on the EuRoc MAV: We test our proposed method on the small-scale and low-resolution EuRoC MAV Dataset [11], which consists of 11 sequences categorized into easy, medium, and difficult classes based on illumination and camera motion. In our method, we did not downsample the sequences from the EuRoC MAV Dataset since they already have a resolution of only 752 × 480 pixels. Additionally, the search radius for the two-step keypoint adjustment is set to 4 pixels.

In Table 2, we provide the $\sigma_{pos}$ results. Given the small scale of the EuRoc sequences, our method shows slight improvements compared with OpenVSLAM [8]. For most sequences, our method delivers either better or comparable results to the state-of-the-art methods. Notably, COLMAP [3] demonstrates competitive accuracy with our method, but our approach is noticeably more efficient, as seen in Table 3. Our method also outperforms Theia [4] in terms of efficiency, except for sequences V102, V103, and V203. However, it is worth noting that, in sequences V103 and V203, Theia exhibits significantly lower accuracy compared with our method.

### 4.3. Ablation Experiment

**Figure 4:**
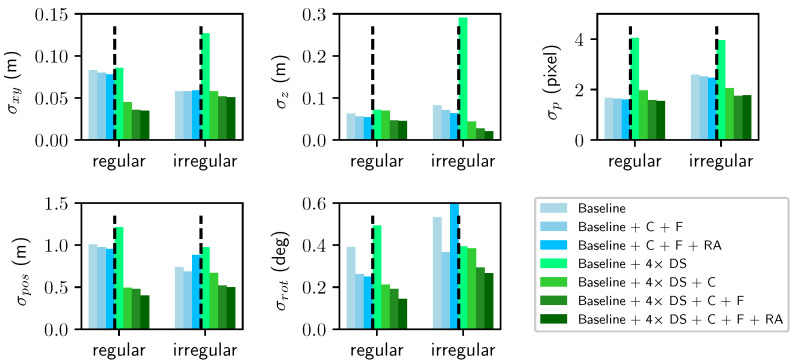
RMSE results. Baseline: bundle adjustment applied once at the original scale, and parameters and keyframes are initialized with OpenVSLAM on 4× downsampled video. DS: downsample; RA: rotation average; BA: global bundle adjustment; C: coarse keypoint adjustment; F: fine keypoint adjustment.

We perform several ablation experiments on the collected dataset. Figure 4 illustrates the results for all metrics under different settings.

TS-KA: as shown in Figure 4, the incorporation of TS-KA enhances accuracy ranging from 2 to 5 times for all metrics when keypoints are extracted from downsampled images. Even when keypoints are obtained in the original scale, TS-KA still enhances matching performance, particularly for $\sigma_{rot}$. This emphasizes the significance of adjusting keypoints prior to global refinement.

Rotation averaging: in the regular sequence, the introduction of global averaged rotations into BA results in a slight improvement for all metrics. However, in the case of the irregular sequence, there can be a slight decrease in accuracy for certain metrics like $\sigma_{pos}$ and $\sigma_{rot}$ on the original scale. This can be attributed to the fact that the view graph of the regular scene is denser, which necessitates the use of rotation averaging. Conversely, in the irregular sequence, the scene may have a sparser view graph, making the global averaged rotations less beneficial.

**Figure 5:**
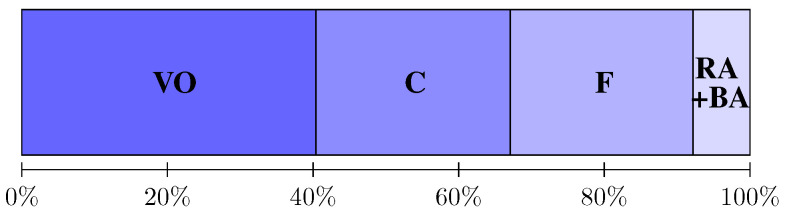
Breakdown timings of each component in regular sequence. RA: rotation averaging regularizer; C: coarse keypoint adjustment; F: fine keypoint adjustment; BA: bundle adjustment.

Accuracy vs precision: Figure 5 shows breakdown timings of each component to the total reconstruction in the regular scene. We see the coarse adjustment takes equal time but gain significantly more improvement in accuracy than fine keypoint adjustment, according to Figure 4. Therefore, it is a good choice to remove the fine adjustment components [20] instead of the coarse adjustment in an efficiency-first scenario.

TS-KA vs FKA [20]: we further compare our TS-KA with the featuremetric keypoint adjustment (FKA) [20] for SfM tasks in six outdoor sequences from the ETH3D benchmark [15]. This benchmark provides ground-truth camera poses, intrinsic parameters, and highly accurate dense point clouds. To evaluate the matching effect, we follow the protocol introduced in [52]. We reconstruct a 3D sparse model using COLMAP [3], with fixed camera intrinsics and poses provided by the authors. We use four different features: SIFT [43], learning-based SuperPoint [45], D2-Net [47], and R2D2 [46] for extracting feature points in the original scale.

The results of applying two keypoint refinement methods on different feature points are presented in Table 4. It can be observed that our method consistently achieves better accuracy and completeness compared with [20] across all feature points in almost all scenes. This consistent improvement confirms that our TS-KA method offers superior keypoint alignment.

**Figure 6:**
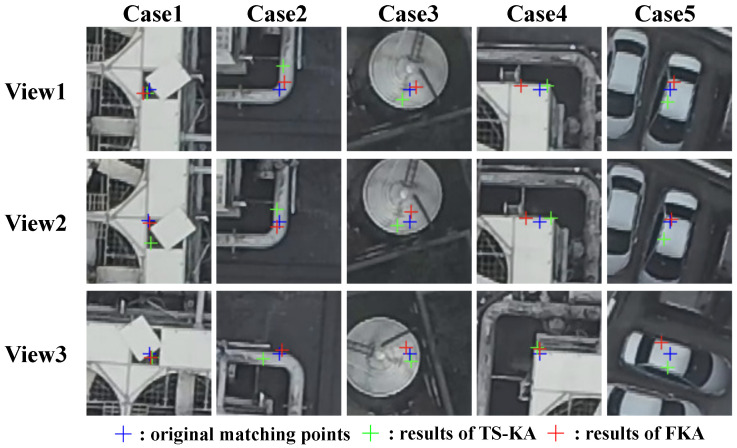
The comparison of keypoint adjustment methods. For each keypoint, we select three views. View 1 and View 2 have similar capture angles, whereas the viewing angle of View 3 varies significantly from them. For each view, we demonstrate the matching positions using different keypoint adjustment methods.

Figure 6 provides samples of feature point refinement, showcasing the ability of our method to adjust feature points in multi-view images to their correct positions. In comparison, FKA [20] is capable of correctly adjusting points under small view angle changes, as demonstrated in the first and second row. However, when faced with significant variations in view angles, as shown in the third row, FKA tends to produce a larger number of incorrect keypoints. This discrepancy largely contributes to the comparatively poorer performance of FKA, as evident in Table 4.

## 5. Conclusions

This paper introduces an efficient SfM pipeline for processing high-resolution, large-size videos. The pipeline utilizes visual odometry to select keyframes and obtain initial camera poses and reconstruction results efficiently by operating on downsampled video data. A two-step keypoint adjustment method, TS-KA, is proposed to efficiently reuse and adjust the keypoints extracted during visual odometry, resulting in improved stability for subsequent global bundle adjustment. Experimental results demonstrate the superior performance and efficiency of our method compared with state-of-the-art SfM and VSLAM methods. Additionally, we have curated and introduced an outdoor high-resolution, large-size video dataset with high-accuracy GCPs, serving as a valuable supplement to existing public video datasets and offering considerable benefits to SfM and VSLAM research.

In this article, we focus exclusively on video-based SfM. With the increasing availability of onboard sensors, we plan to integrate our method with data from other sensors, such as IMU and GNSS, to further enhance the accuracy and robustness of our algorithm.

## Figures and Tables

**Table 1:** Trajectory error metrics and efficiency on our dataset. $\delta_{xy}$ (cm), $\delta_{z}$ (cm), $\delta_{p}$ (pixel): RMSE error in (8). $\delta_{pos}$ (m), $\delta_{rot}$ (deg): RMSE error in (9). The 3840 × 2160 videos are 4× downsampled only on our method. ‘*’ indicates models are initialized using per second sampling.

Method	w/ GCPs			w/o GCPs		
	$\sigma_{\mathrm{xy}}$	$\sigma_{z}$	$\sigma_{p}$	$\sigma_{\mathrm{pos}}$	$\sigma_{\mathrm{rot}}$	Time (s)
**(a) Regular sequence**						
COLMAP [3]	6.8	7.4	1.78	1.99	0.34	-
COLMAP * [3]	6.9	12	1.81	2.14	1.04	4650
Theia [4]	8.8	8.9	1.93	1.32	0.46	-
Theia * [4]	10	25	2.86	2.30	1.15	1413
OpenVSLAM [8]	8.3	6.3	1.67	1.10	0.39	1812
Ours	**3.5**	**4.6**	**1.55**	**0.40**	**0.14**	**632**
**(b) Irregular sequence**						
COLMAP [3]	10	13	3.12	0.64	0.52	-
COLMAP * [3]	11	11	3.95	1.87	1.83	550
Theia [4]	6.6	6.2	1.97	1.33	1.08	-
Theia * [4]	10	9	3.55	1.56	1.47	648
OpenVSLAM [8]	5.8	8.3	2.59	0.74	0.53	430
Ours	**5.1**	**2.1**	**1.77**	**0.50**	**0.27**	**316**

Bold represents the optimal metrics.

**Table 2:** $\sigma_{pos}$ on EuRoc MAV [11]. The 752 × 480 EuRoc sequences are not downsampled in our method.

Method	M01	M02	M03	M04	M05	V101	V102	V103	V201	V202	V203	Mean
COLMAP [3]	**0.037**	0.033	0.052	**0.073**	0.053	**0.089**	**0.063**	0.088	0.064	0.056	**0.058**	0.061
Theia [4]	0.040	0.033	0.072	0.269	0.078	0.091	0.067	0.156	0.072	0.088	1.980	0.267
OpenVSLAM [8]	0.041	**0.032**	0.033	0.096	0.049	0.096	0.064	0.066	0.061	0.053	0.072	0.060
Ours	0.040	**0.032**	**0.032**	0.093	**0.048**	0.094	**0.063**	**0.065**	**0.059**	**0.053**	0.071	**0.059**

Bold represents the optimal metrics.

**Table 3:** A comparison of processing time (s) on EuRoc MAV [11]. ‘*’ indicates that in this scene Theia produces a very rough trajectory.

Method	M01	M02	M03	M04	M05	V101	V102	V103	V201	V202	V203
COLMAP [3]	534	463	279	187	226	371	63	254	202	144	564
Theia [4]	148	131	108	81	68	127	**50**	**41** *	85	85	**25** *
Ours	**132**	**105**	**65**	**71**	**58**	**101**	63	55	**78**	**63**	65

Bold represents the optimal metrics.

**Table 4:** Results of 3D sparse reconstruction using our TS-KA or FKA [20] on different feature point extractors. We use metrics “accuracy” and “completeness” for threshold 1 cm, 2 cm, and 5 cm, as defined in [55].

Features Refinement	ETH3D Outdoor					
	Accuracy (%)			Completeness (%)		
	1 cm	2 cm	5 cm	1 cm	2 cm	5 cm
SIFT [43]	62.36	71.70	86.27	0.06	0.34	2.65
FKA [20]	65.63	76.25	91.19	**0.07**	**0.40**	2.86
Ours	**66.48**	**78.75**	**92.12**	**0.07**	**0.40**	**2.90**
SuperPoint [45]	49.19	64.34	82.74	0.09	0.49	3.46
FKA [20]	67.20	79.84	90.63	0.16	0.82	**4.98**
Ours	**68.17**	**80.13**	**90.87**	**0.17**	**0.83**	4.96
D2-Net [47]	34.66	51.38	72.12	0.02	0.13	1.77
FKA [20]	64.68	79.17	90.88	0.08	**0.59**	**5.37**
Ours	**65.39**	**80.18**	**91.25**	**0.09**	**0.59**	5.36
R2D2 [46]	42.71	59.81	80.71	0.05	0.36	3.02
FKA [20]	64.02	77.77	90.19	**0.11**	0.60	4.01
Ours	**64.19**	**77.99**	**90.24**	**0.11**	**0.61**	**4.04**

Bold represents the optimal metrics.

## Data Availability

The data presented in this study will be available at http://gpcv.whu.edu.cn/data/WHU_Areial_Video_Dataset.html (accessed on 2 April 2024).
